# Characterisation of two unique sesquiterpenoids from *Trichoderma hypoxylon*

**DOI:** 10.1080/21501203.2021.1964630

**Published:** 2021-08-12

**Authors:** Jinyu Zhang, Wen-Bing Yin

**Affiliations:** a State Key Laboratory Of Mycology, Institute Of Microbiology, Chinese Academy Of Sciences, Beijing, Republic of China; b University Of Chinese Academy Of Sciences, Beijing, Republic of China

**Keywords:** Sesquiterpenes, fungi, *trichoderma*, glycoside, rearranged cuparane

## Abstract

Two new sesquiterpenoids, **1**–**2**, together with three known compounds, were isolated from *Trichoderma hypoxylon*. Among the known compounds, compound **4** was isolated as naturally occurring compound for the first time. The structures of these new compounds were characterized by HR-ESI-MS and spectroscopic methods including 1D and 2D NMR. The absolute configurations of **1**–**2** were assigned by electronic circular dichroism (ECD) calculations.

## Introduction

Trichothecene is a family of sesquiterpenoid mycotoxins and widely found in nature, which is one of the six major classes of frequently occurring mycotoxins (Nielsen et al. 2005; McCormick et al. 2011; Proctor et al. 2018). Due to their well-documented inhibition of protein synthesis and their serious impact on grain production and human health, trichothecenes were of great concern (Desjardins 2009; McLaughlin et al. 2009; Malmierca et al. 2013; Shentu et al. 2018; Ueno 1985). *Trichoderma* spp. produce a large number of trichothecenes, which was widely used in agriculture biological prevention (López et al. 2019; Loc et al. 2020). In the previous works, secondary metabolites of *T. hypoxylon* was studied, while the trichothecenes, epidithiodiketopiperazine alkaloids, amphiphilic coprogens were isolated (Li et al. 2016; Liu et al. 2021; Sun et al. 2016; Zhang et al. 2021). Meanwhile, two polycyclic lactones, tricholactone A and B, and three new sesquiterpenes, tricinoloniol acids A-C, were obtained by the strategy of genetic dereplication (Chen et al. 2019; Liu et al. 2020). It is noteworthy that *T. hypoxylon* produced an abundance of structurally diverse trichothecenes including harzianum B as the major compound (Li et al. 2016; Liu et al. 2018). Harzianum B had significant plant toxicity on both dicot and monocot, which had development potential as herbicide (Yin et al. 2020). At the same time, harzianum B was potential anticancer drug lead with advanced development (Jarvis et al. 1980, 1984; Jin et al. 2007). It is necessary to elucidate the biosynthetic pathway of harzianum B. In order to find the intermediates of harzianum B, we further investigated secondary metabolites of *T. hypoxylon*. In this study, fractionation of the extract of *T. hypoxylon* afforded two new sesquiterpenes including one rare rearranged cuparane-type sesquiterpenoid trihypodione A (**1**), and a cuparane sesquiterpene glycoside, dunnianoside B (**2**), together with three known compounds including FS-3 (**3**), compound **4**, and chartarene A (**5**) (Sanson et al. 1989; Sacks et al. 2015; Li et al. 2017). Remarkably, compound **4** was isolated from nature for the first time. Herein, we described the isolation and structural determination of two new sesquiterpenes **1** and **2**.

## Materials and methods

### General experimental procedures

Analytical grade solvents were used for extraction and chromatographic separation. Silica gel (200–300 mesh, YanTai JiangYou Chemical Co., Ltd., China) and octadecyl silane (ODS) (45–70 µm, Merck, Darmstadt, Germany) were used for column chromatography. Analytical HPLC was conducted with a Waters HPLC system (Waters e2695, Waters 2998, Photodiode Array Detector) using a column chromatography, ODS (YMC-Pack ODS-A, 10 × 250 mm, 5 µm, detector: UV) column with a flow rate of 1.0 mL/min. HPLC separation was carried out on SSI HPLC instrument (Scientific Systems Inc., Pennsylvania, USA), using a YMC-Pack ODS-A column (20 × 250 mm, 5 µm, detector: UV) with a flow rate of 2.0 mL/min. The optical rotations were taken on a PerkinElmer 241 polarimeter, while the CD spectra were recorded on a JASCO J-815 spectrometer. UV spectra were measured on a ThermoGenesys-10S UV-vis spectrophotometer. IR spectra were obtained on a Nicolet IS5FT-IR spectrophotometer. Nuclear magnetic resonance (NMR) spectra were recorded on a Bruker Avance-500 spectrometer at room temperature (Bruker Corporation, Karlsruhe, Germany). HR-ESI-MS data were recorded on an Agilent Technologies 6520 Accurate-Mass Q-TOF LC/MS spectrometer equipped with an electrospray ionisation (ESI) source.

### Strain

***Trichoderma**hypoxylon* was deposited in the China General Microbiological Culture Collection Center (CGMCC 3.17906) (Sun et al. 2016).

#### Extraction and isolation

***Trichoderma**hypoxylon* was grown on PDA plates at 25°C for 5 days and then inoculated into PDB medium to incubation for 5 days at 25°C on a rotary shaker at 200 rpm. Then, each 20 mL seed was transferred into a 500 mL Erlenmeyer flask containing 100 g rice and 120 ml distilled H_2_O for large-scale fermentation. After incubated at 25°C for 40 days, the fermented product was extracted successively with 20 L ethyl acetate at room temperature and the organic phase layer was evaporated to dryness under vacuum to yield the crude extract (80.4 g). The crude extract was subjected to silica gel column chromatography, eluting with dichloromethane and acetone in a gradient manner (v/v, 20:80, 40:60, 50:50, 75:25, 100:0) to obtain 21 fractions named fraction T1 to T21. T13 (8.5 g) was separated by ODS chromatography eluting with methanol and H_2_O (v/v, 20:80, 30:70, 40:60, 50:50, 70:30, 90:10, 100:0) to give 15 fractions named fraction T13–1 to T13–15. The compounds **1** (3 mg) and **3** (4 mg) were purified from T13–6 (75.4 mg) by a semipreparative HPLC (28:72 CH_3_CN/H_2_O, 2 mL/min), while the compound **5** (6 mg) were obtained from T13–5(33:67 CH_3_CN/H_2_O, 2 mL/min). The compound **2** (4 mg) were purified from T15 (35 mg) by semipreparative HPLC (44:56 CH_3_CN/H_2_O, 2 mL/min). Further T16 was separated by a ODS column with methanol-H_2_O to give 18 fractions named fraction T16–1 to T16–18. Then the compound **4** (5 mg) was obtained from T16–16 by semi-preparative HPLC (45:55 CH_3_CN/H_2_O, 2 mL/min).

Trihypodione A (**1**). White amorphous powder (methanol); [α]^25^_D_ +54.99 (*c* 1.5, methanol); CD (*c* 1.5 × 10^−3^ M, methanol) *λ*_max_ (Δ*ε*): 226 (+17.02), 250 (−5.81), 311 (+0.99); UV (methanol) *λ*_max_ nm (log *ε*): 240 (4.02); IR (neat) *ν*_max_: 3443,2962, 1668, 1260, 1032, 800 cm^−1^. For ^1^H NMR and ^13^C NMR data see Table 1. Positive HR-ESI-MS: *m/z* [M + H]^+^ 249.1408 (calcd. for C_15_H_21_O_3_ 249.1412).

**Table 1.** NMR spectroscopic data for **1**–**2** in methanol-*d*_4._

No.	**1**		**2**	
	*δ*_C_^a^	*δ*_H_ (mult., *J* in Hz)^b^	*δ*_C_^a^	*δ*_H_ (mult., *J* in Hz)^b^
1	186.4	-	48.5	-
2	133.4	6.16 (s)	54.4	-
3	210.2	-	222,.3	-
4	50.5	2.13 (d 18.5), 2.67 (d 18.5)	75.5	4.60 (dd 9.5, 2.2)
5	52.2	-	39.1	2.03 (dd 14.5, 2.2), 3.20 (dd 14.6, 9.7)
6	50.2	-	142.2	-
7	37.4	1.98(m), 2.51 (m)	127.8	7.30 (d 8.1)
8	29.6	2.29(m), 2.43 (m)	129.8	7.17 (d 8.0)
9	165.2	-	137.0	-
10	127.8	5.93 (s)	129.8	7.17 (d 8.0)
11	203.0	-	127.8	7.30 (d 8.1)
12	67.7	3.55 (d 10.7), 3.75 (d 10.7)	22.7	0.67 (s)
13	24.3	1.13 (s)	18.9	1.17 (s)
14	25.7	1.39 (s)	27.0	1.39 (s)
15	24.0	1.98 (s)	20.9	2.32 (s)
1′			101.0	5.30 (d 3.7)
2′			73.6	3.43(dd 9.8, 3.8)
3′			74.8	3.66 (m)^c^
4′			71.9	3.29 (m)
5′			74.6	3.66 (m)^c^
6′			62.9	3.69 (d 6.1), 3.86 (d 10.6)

^a^Recorded at 500 MHz. ^b^ Recorded at 125 MHz. ^c^These signals are overlapped.

Dunnianoside B (**2**). White amorphous powder (methanol); [α]^25^_D_ +89.98 (*c* 1.2, methanol); CD (*c* 1.2 × 10^−3^ M, methanol) *λ*_max_ (Δ*ε*): 227 (−4.96), 307 (+4.99); UV (methanol) *λ*_max_ nm (log *ε*): 196 (4.20), 219 (3.04); IR (neat) *ν*_max_: 3376, 2968, 1741, 1379, 1028, 527 cm^−1^. For ^1^H NMR and ^13^C NMR data see Table 1. Positive HR-ESI-MS: *m/z* [M + H]^+^ 395.1995 (calcd. for C_21_H_31_O_7_ 395.1992).

#### Acid hydrolysis of 2 and analyses

Compound **2** (1.5 mg) was hydrolysed with 2 mL 2 M HCl at 90°C for 2 h. The mixture was evaporated and resuspended in 1 mL H_2_O and extracted with ethyl acetate. After added with 1 mL pyridine and 2.5 mg L-cysteine methyl ester into the water layer and incubate at 60°C for 2 h, the reaction mixture was added with 5 µL o-tolyl isothiocyanate and further heated at 60°C for another 1 h. The mixtures were analysed by HPLC with a UV detector (250 nm). The mobile phase consisted of 25% acetonitrile and 75% H_2_O with 0.1% formic acid. D-galactose, D-fructose, D-glucose and L-glucose, using as sample, were derivatised using L-cysteine methyl ester and o-tolyl isothiocyanate in the same procedure (Tanaka et al. 2007).

#### Computation section

The MMFF94 molecular mechanics force field were used for systematic conformational analysis of **1a** and **6a**. Subsequently, the conformers were obtained and then optimised with Gaussian software package at the B3LYP/6–31 G(d, p) basis set level using density-functional theory (DFT). The stationary points were also checked as the true minima of the potential energy surface by verifying they do not exhibit vibrational imaginary frequencies. The 40 lowest electronic transitions were calculated using time-dependent density-functional theory (TDDFT) methodology at the B3LYP/6–31 G(d, p) lever. ECD spectra were stimulated using a Gaussian function with a half-bandwidth of 0.40 and 0.45 eV, respectively. The overall ECD spectra were then generated according to Boltzmann weighting of each conformer.

## Result

**Figure 1.**
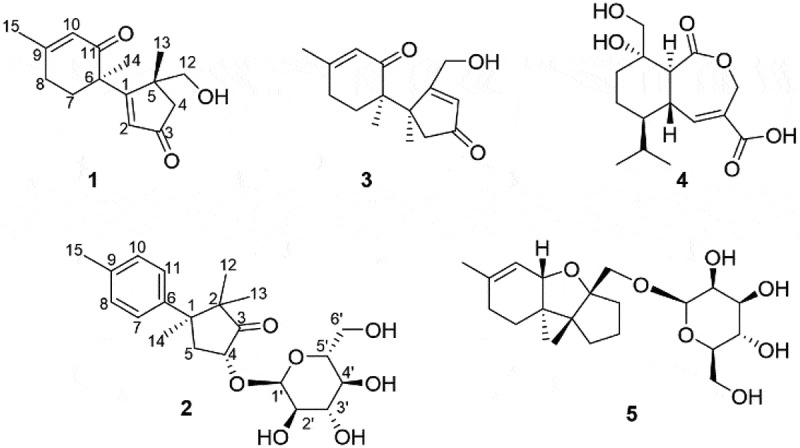
Structures of trihypodione A (**1**), dunnianoside B (**2**), **3**, **4** and **5**.

***Trichoderma** hypoxylon* was cultured on rice medium for 40 days. The media and mycelia were extracted with ethyl acetate, and the organic extracts were subsequently subjected to a combination of silica gel column chromatography, sephadex LH-20 column chromatography, ODS and semipreparative HPLC to yield compounds **1**–**5 (Figure 1)**.

Trihypodione A (**1**) was isolated as white amorphous powder. It had a molecular formula of C_15_H_20_O_3_ as determined by HRESIMS ion at *m/z* 249.1408 [M + H]^+^, containing 6 degrees of unsaturation. The ^1^H, ^13^C NMR and HSQC data (Table 1) suggested the presence of three methyl groups (*δ*_C_ 24.0, 24.3 and 25.7), four methylene groups including one oxygenised methylene (*δ*_C_ 67.7), two sp^3^-hybridised quaternary carbons (*δ*_C_ 50.2 and 52.2), four olefinic carbons (*δ*_C_ 127.8, 133.4, 165.2, and 186.4), and two ketone carbonyl carbons (*δ*_C_ 203.0 and 210.2).

**Figure 2.**
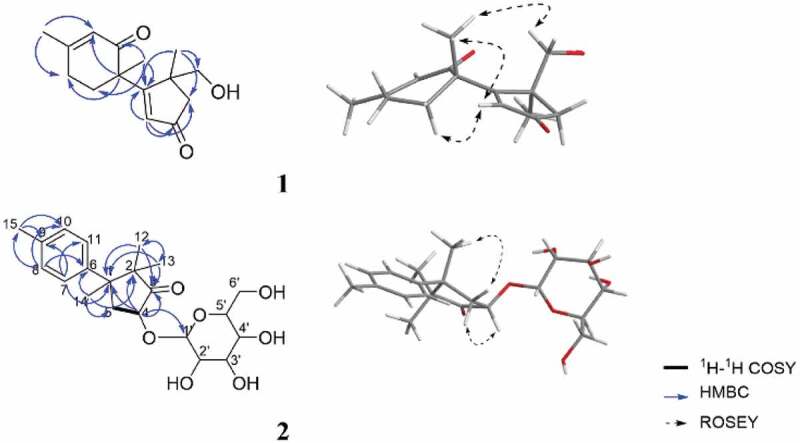
Selected key ^1^H-^1^H COSY, HMBC and NOESY correlations of **1** and **2**. the molecular models of **1** and **2** in minimal energy were obtained by conflex calculations in MMFF94s force field.

**Figure 3.**
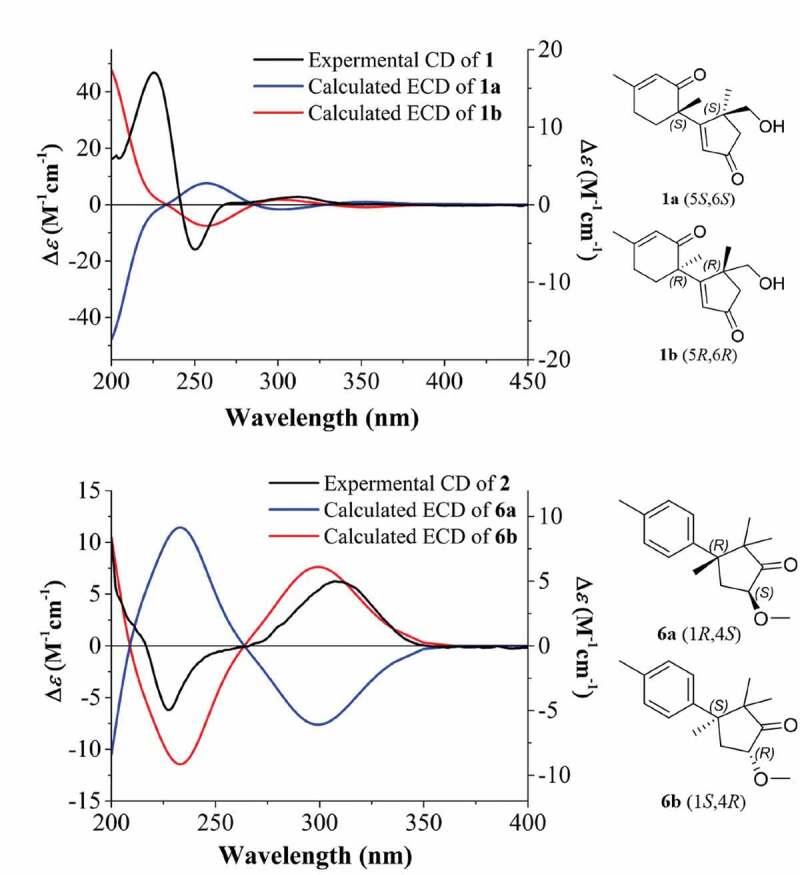
Experimental CD spectra of **1** and **2** in methanol and the calculated ECD spectra of **1a**, **1b**, **6a**, and **6b**. structures **1a**, **1b**, **6a**, and **6b** represent possible stereoisomers for **1** and **2**, respectively.

In the HMBC spectrum of **1**, the correlations from H_3_–13 to C-1, C-4, C-5 and C-12, from H_2_–12 to C-1, C-4, C-5 and C-13, from H_2_–4 to C-3 and from H-2 to C-1, C-3, and C-4 furnished an *α, β*-unsaturated cyclopentanone moiety, also showed that the methyl group at C-13 and the hydroxyl group at C-12 were attached to C-5. The HMBC correlations from H-7 to C-8, C-9 and C-11, from H-10 to C-6, C-8 and C-11, and from H_3_–15 to C-8, and C-10 confirmed the presence of 3-methylcyclohex-2-en-1-one. Further inspection of the HMBC correlations from H-2 to C-6, from H_3_–14 to C-1, C-7, and C-11 not only connected C-1 with C-6 but also established the planar structure and satisfied the unsaturation requirement (Figure 2). The relative configuration of **1** was determined as shown by ROESY correlations of H-2 with H-7 with H_3_–14, as well as H_2_–12 with H_3_–14 (Figure 2). By comparison of the experimental and simulated ECD curves (Figure 3), the absolute configurations at C-5 and C-6 were assigned as 5 *R*,6 *R* in **1**.

Dunnianoside B (**2**) was obtained as white powder with the molecular formula C_21_H_30_O_7_ as determined by HR-ESI-MS 395.1995 [M + H]^+^, indicating 7 degrees of unsaturation. The ^1^H, ^13^C NMR and HSQC data (Table 1) suggested the presence of four methyls (*δ*_C_ 18.9, 20.9, 22.7, and 27.0), one methylene, two sp^3^-hybridised quaternary carbons (*δ*_C_ 48.5 and 54.4), six olefinic carbons (*δ*_C_ 127.8, 129.8, 137.0 and 142.2), and one ketone carbonyl carbon (*δ*_C_ 222.3). The remaining six oxygenised carbons (*δ*_C_ 101.0, 74.8, 74.6, 73.6, 71.9, 62.9) indicated the characteristic resonances for a glucopyranose fragment. The ^1^H-^1^H COSY correlations of H-4 to H_2_–5 and, in the HMBC spectrum, the correlations from H_3_–13 to C-1, C-3 and C-12, from H_3_–12 to C-1, C-3, and C-13, from H_2_–4 to C-1, C-2, and C-3, and from H_3_–14 to C-2, and C-5 furnished a 2,2,3-trimethylcyclopentan-1-one moiety, also showed that the methyl group at C-12 and C-13 were attached to the C-2. The HMBC correlations from H_3_–15 to C-9, and C-10, from H-8 to C-6, C-10, and C-15, and from H-7 to C-9, and C-11. Further analysis of HMBC correlations from H-7 to C-1, from H-14 to C-6 connected C-1 with C-6 established the planar structure as shown in Figure 2. Acid hydrolysis and derivation of **2** confirmed the presence of D-glucose by HPLC analysis. The smaller coupling constant (3.7 Hz) suggested the α configuration for the anomeric carbon of the glucose, which was attached to C-4 in **2**.

A detailed analysis of the ROESY spectrum of two showed the correlations of H-4 with H_3_–12 and H-5a (*δ*_H_ 3.20) together with H_3_–14 with H-5b (*δ*_H_ 2.00) and H_3_–13 (Figure 2). Considering the complicated conformation of the flexible glucopyranose fragment and its insignificant effect on the CD spectrum, compound **2** was simplified as structure **6** for CD calculation. The absolute configuration of **2** was deduced as 1*S*,4*R* by comparison of the experimental and simulated ECD spectra (Figure 3).

## Discussion

Trichothecenes could be divided into two main categories: the simple trichothecenes and the macrocyclic trichothecenes (Type D trichothecenes), while macrocyclic trichothecenes had more potential as antitumor agents compared with the simple trichothecenes (de Carvalho et al. 2016; Proctor et al. 2018; Smitka et al. 1984). Harzianum B was an important precursor of macrocyclic trichothecenes, for which biosynthesis was not integrally elucidated, especially the connection method of macrocycle (Proctor et al. 2020; Zhu et al. 2020). In this work, two new sesquiterpenes **1**–**2**, together with three known compounds, were isolated and identified. Structurally, these compounds were intermediates or shunt products of the harzianum B biosynthesis pathway. Of note, both of compounds **2** and **5** contained glycosyl groups, while compound 1 had a special methyl transfer reaction compared to compound **3**. These phenomena indicated that there may exist a glycosyltransferase and a special methyltransferase in the biosynthesis pathway of trichothecenes, which play certain roles in the structural diversity of trichothecenes. The yield of compounds **1**–**5** are low in nature. In order to better study their roles in the biosynthesis of harzianum B, synthetic biology methods or gene regulation approaches can be used to increase their yields (Lyu et al. 2020).

In summary, we described two new sesquiterpenes, together with three known compounds. The discovery of two new intermediates may provide the basis for a more comprehensive analysis of the biosynthetic pathway of macrocyclic trichothecenes.

## Data Availability

The data used to support the findings of this study are available from the corresponding author upon request.
